# Prevalence and risk factors for dementia and mild cognitive impairment among older people in Southeast China: a community-based study

**DOI:** 10.1186/s12877-024-05054-6

**Published:** 2024-05-28

**Authors:** Bin Jiang, Qi Liu, Jian-Peng Li, Si-Ning Lin, Hui-Juan Wan, Zi-Wen Yu, Jing Wang, Wei Zhuang, Jia-Hui Tang, Cai-Hong Chen, Fa-Yin Li, Min Bi, Nai-An Xiao, Kun-Mu Zheng

**Affiliations:** 1 Department of Neurology and Department of Neuroscience, The First Affiliated Hospital of Xiamen University, School of Medicine, Xiamen University, Xiamen, China; 2 The School of Clinical Medicine, Fujian Medical University, Fuzhou, China; 3 Department of Neurology, Nanjing County Hospital, Zhangzhou, China; 4 Department of Neurology, The Third Hospital of Xiamen, Xiamen, China; 5 Xiamen Key Laboratory of Brain Center, Xiamen, China

**Keywords:** Dementia, Mild cognitive impairment, Prevalence, Risk factors, Chinese older adults

## Abstract

**Background:**

With the aging population, the number of individuals with dementia in China is increasing rapidly. This community-based study aimed to investigate the prevalence and risk factors for dementia and mild cognitive impairment (MCI) among older adults in China.

**Methods:**

In this study, 20,070 individuals aged ≥ 65 were recruited between January 1, 2022, and February 1, 2023, from ten communities in Xiamen City, China. We collected data on age, sex, level of education, and medical history, as well as global cognition and functional status. The prevalence of dementia and MCI was examined, and the risk factors for different groups were assessed.

**Results:**

The overall prevalence of dementia and MCI was approximately 5.4% (95% confidence interval [CI], 5.1–5.7) and 7.7% (95% CI, 7.4–8.1), respectively. The results also indicated that dementia and MCI share similar risk factors, including older age, female sex, hypertension, and diabetes mellitus. Compared with individuals with no formal education, those with > 6 years of education had an odds ratio for MCI of 1.83 (95% CI, 1.49–2.25). We also found that only 5.5% of the positive participants chose to be referred to the hospital for further diagnosis and treatment during follow-up visits.

**Conclusions:**

This study estimated the prevalence and risk factors for dementia and MCI among individuals aged ≥ 65 years in Southeast China. These findings are crucial for preventing and managing dementia and MCI in China.

## Background

Dementia, a common neurocognitive disorder with an insidious onset, is characterized by chronic and progressive cognitive impairment, accompanied by a decline in the ability to perform activities of daily living [1]. With an increasing aging population, the number of individuals with dementia is rapidly growing, and more than 150 million individuals worldwide are projected to have dementia by 2050 [2]. In China, approximately 10 million people currently live with dementia, and the socioeconomic cost associated with it is projected to reach $250 billion by 2020 [3]. The burden of dementia poses significant financial, social, and public health challenges worldwide. Mild cognitive impairment (MCI) is considered to be a stage between normal physiological aging and dementia, potentially progressing to dementia within 5–10 years [4]. Effective therapies for dementia are currently lacking, emphasizing the importance of primary prevention [5]. MCI is a critical interventional target for dementia. Consequently, a better understanding of MCI, including early identification, underlying etiology, risk factor definition, and available pharmacological and non-pharmacological treatments, has become an important research topic [6, 7].

Several studies have investigated the prevalence of dementia and MCI in the Chinese population. However, the results indicated significant variations across various regions of China. The findings of a cross-sectional national survey involving Chinese individuals aged ≥ 60 years indicated a prevalence of 6.0% for dementia and 15.5% for MCI [8]. Another study found the prevalence of dementia and MCI to be 8.04% and 18.76%, respectively, among Chinese individuals aged ≥ 65 years [9]. A meta-analysis reported that the prevalence of dementia in Northern China (5.4%) was higher than that in central (3.8%) and Southern China (3.7%) [10]. Furthermore, a systematic review revealed that the prevalence of MCI in Eastern China (13.14%) was lower than that in Western China (14.33%) [11]. Additionally, the risk factors for dementia have evolved over the past decade, influenced by environment, urbanization, and lifestyle changes. A previous analysis indicated that unhealthy lifestyles, including high fat intake and physical inactivity, contribute to an increased incidence of cognitive impairment [12].

In our clinical practice, we found that over 50% of the patients exhibited moderate-to-severe dementia during their initial hospital visit due to a lack of early screening [13]. Early screening and health education within communities play important roles in the early identification of dementia in older people, especially in those with limited mobility and insufficient care [14]. In community-based screening through home visits, trained professionals, such as physicians or nurses, can provide a brief cognitive assessment for the early identification of cognitive decline and improve the knowledge of preventing dementia in older adults. This study investigated the prevalence and risk factors for dementia and MCI in a large sample of individuals aged ≥ 65 years from 10 communities in Southeast China.

## Methods

### Participants and preparation procedure

In this cross-sectional study, we recruited participants aged ≥ 65 in Xiamen City between January 1, 2022, and February 1, 2023. We randomly selected two communities from each district (a total of ten communities) in Xiamen City. Prior to the interviews, we contacted all older people aged ≥ 65 years in this community by telephone, facilitated by community volunteers, to inquire if they were willing to participate in our survey. Subsequently, we conducted home visits to those who agreed to participate and conducted relevant interviews after obtaining signed informed consent. Four to five teams of interviewers were formed for each community, each comprising one community doctor, one community nurse, and one community staff member. Furthermore, we established a community expert panel comprising two neurologists and two neuropsychologists with expertise in cognitive impairment disorders. The expert panel and interviewers reviewed all data and assigned final diagnoses. The interviewers underwent a week-long training course covering knowledge and diagnosis of dementia and MCI, as well as assessment procedures for cognitive measurement tools. The inter-rater reliability for cognitive diagnoses and assessments needed to exceed 0.90. The expert panel conducted logic checks for inconsistencies and audits to ensure the quality of the entered data. Thus, all interviews were conducted by well-trained interviewers, with good quality control measures. The in-person interviews with the participants or their guardians were conducted at their residences through home visits. Information regarding age, sex, education level, and medical history was collected using a structured questionnaire. Thereafter, cognitive evaluations were conducted for the participants, including the Mini-Mental State Examination (MMSE) and the Clinical Dementia Rating (CDR). The functional status was assessed using the Basic Activities of Daily Living and Instrumental Activities of Daily Living scales [15]. Meanwhile, we brought portable blood pressure and blood glucose monitors to measure the participants’ blood pressure and random blood glucose levels, respectively, before the interview. Combined with medical records and testing results, when doubts about the diagnosis arose, we conducted fasting blood glucose and blood pressure exams one or two times on different days during home visits to confirm the diagnosis. Written informed consent was obtained from the participants themselves or their guardians. The study was approved by the Research Ethics Committee of the First Affiliated Hospital of Xiamen University.

### Diagnosis criteria

Participants were categorized into groups representing normal cognitive function, MCI, or dementia. Normal cognitive function was defined when participants have a CDR score of 0 as well as an MMSE score > 19 for illiterate individuals, > 22 for individuals with 1–6 years of education, and > 26 for individuals with > 6 years of education. Dementia was defined when participants have a CDR score ≥ 1 as well as an MMSE score ≤ 17 for illiterate participants, ≤ 20 for participants with 1–6 years of education, and ≤ 24 for participants with > 6 years of education [16]. MCI was determined when participants have a CDR score of 0.5 as well as an MMSE score ≤ 19 for illiterate participants, ≤ 22 for participants with 1–6 years of education, and ≤ 26 for participants with > 6 years of education [17].

### Statistical analysis

The prevalence of dementia or MCI, along with the 95% confidence interval (CI), was calculated for all participants and subgroups based on age, sex, and educational level. A multivariable logistic regression model was employed to identify the risk factors for dementia or MCI, including age (65–74, 75–84, and ≥ 85 years), sex, educational level, and presence of hypertension or diabetes mellitus (DM). Multivariable adjusted odds ratios (ORs), with corresponding 95% CIs, were reported. All statistical analyses were conducted using SPSS 25.0 (IBM Corp., Armonk, N.Y., USA), using two-sided statistical tests. Statistical significance was set at *p* < 0.05.

## Results

A total of 21,978 participants aged ≥ 65 years from 10 communities in Xiamen City were invited to participate in this study. Among them, 1,908 were excluded: 1,460 had incomplete or doubtful data, 293 had hearing or vision loss, and 155 withdrew their consent. Consequently, 20,070 participants were included in the final analysis. The demographic and clinical characteristics of the participants are shown in Table 1. The mean age was 73.14 ± 6.39 years, and 9,719 participants (45.7%) were males. Notably, 1,960 participants (9.8%) were uneducated, and 4,969 (24.8%) had ≤ 6 years of education. Among the 20,070 participants, we identified 1,082 (5.4%) with dementia and 1,553 (7.7%) with MCI.

**Table 1:** Demographics and clinical characteristics of the study population by cognitive status

	Total sample (*n* = 20,070)	Cognitively normal (*n* = 17,435)	MCI (*n* = 1,553)	Dementia (*n* = 1,082)	*p* value
Age, mean (SD) years	73.14 (6.39)	72.72 (5.99)	74.09 (6.73)	78.59 (9.01)	< 0.001
Age group, n (%)					< 0.001
65–74 years	13,175 (65.6)	11,874 (68.1)	880 (56.7)	421 (38.9)	
75–84 years	5,594 (27.9)	4,704 (27.0)	548 (35.3)	342 (31.6)	
≥ 85 years	1,301 (6.5)	857 (4.9)	125 (8.0)	319 (29.5)	
Gender, n (%)					< 0.001
Male	9,179 (45.7)	8,082 (46.4)	686 (44.2)	411 (38.0)	
Female	10,891 (54.3)	9,353 (53.6)	867 (55.8)	671 (62.0)	
Educational level, n (%)					< 0.001
< 1 years	1,960 (9.8)	1,679 (9.6)	109 (7.0)	172 (15.9)	
1–6 years	4,969 (24.8)	4,425 (25.4)	264 (17.0)	280 (25.9)	
> 6 years	13,141 (65.6)	11,331 (65.0)	1,180 (76.0)	630 (58.2)	
Hypertension, n (%)					< 0.001
No	11,094 (55.3)	9,989 (57.3)	703 (45.3)	402 (37.2)	
Yes	8,976 (44.7)	7,446 (42.7)	850 (54.7)	680 (62.8)	
Diabetes, n (%)					< 0.001
No	16,187 (80.7)	14,296 (82.0)	1,171 (75.4)	720 (66.5)	
Yes	3,883 (19.3)	3,139 (18.0)	382 (24.6)	362 (33.5)	
MMSE score, mean (SD)	27.09 (4.11)	28.04 (2.13)	24.49 (2.26)	15.45 (8.57)	< 0.001
ADLs score	21.84 (6.08)	20.04 (0.22)	25.26 (1.88)	45.89 (6.33)	< 0.001
CDR stage	0.16 (0.54)	0	0.5	2.23 (0.72)	< 0.001

Note: MCI, mild cognitive impairment; SD, standard deviation; MMSE, mini-mental state examination; ADL, Activities of Daily Living; CDR, Clinical Dementia Rating; ADLs score: the Basic ADL score (8–32) + the Instrumental ADL score (12–48)

**Fig. 1:**
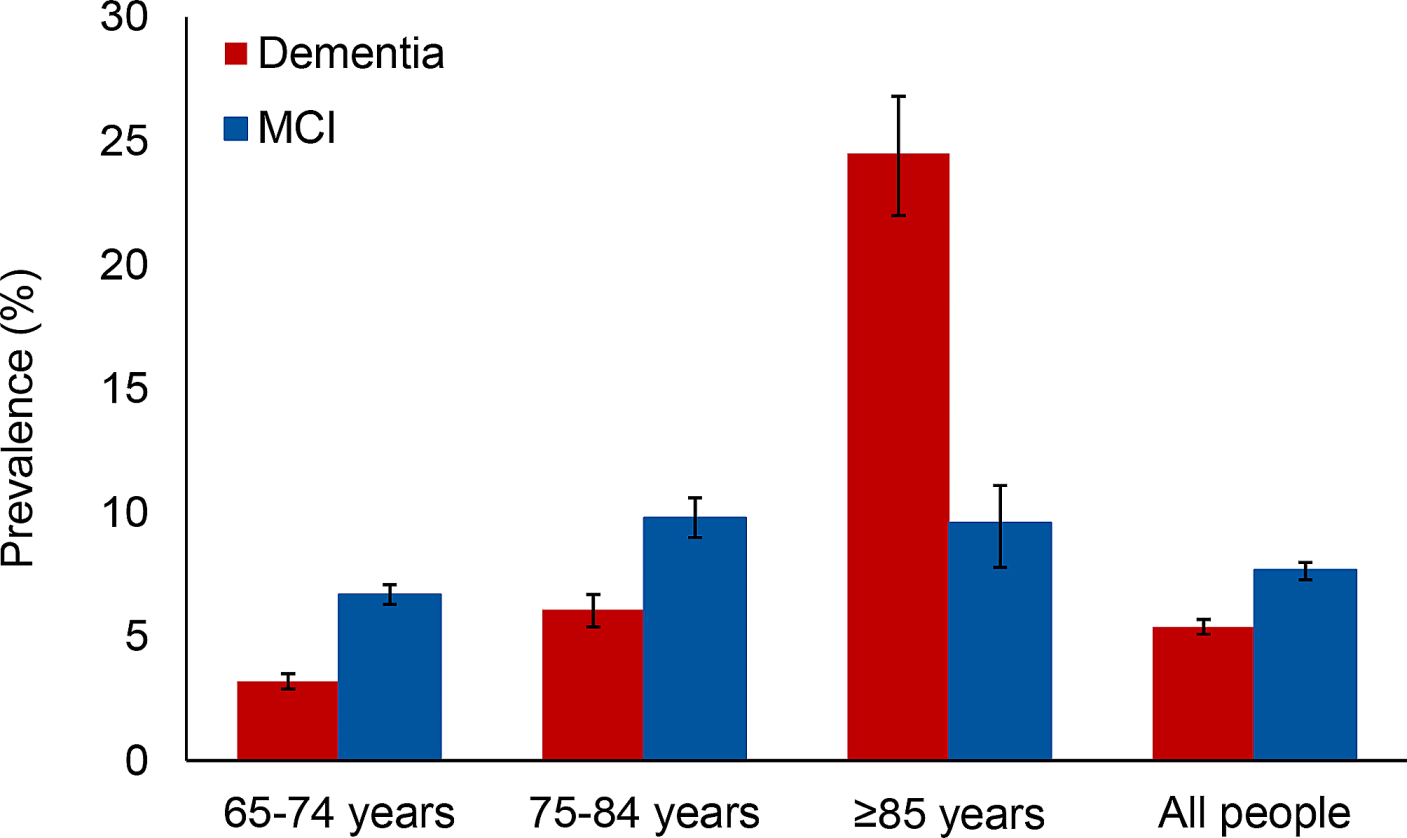
Prevalence of dementia and mild cognitive impairment among people aged ≥ 65 years, stratified by age group

**Fig. 2:**
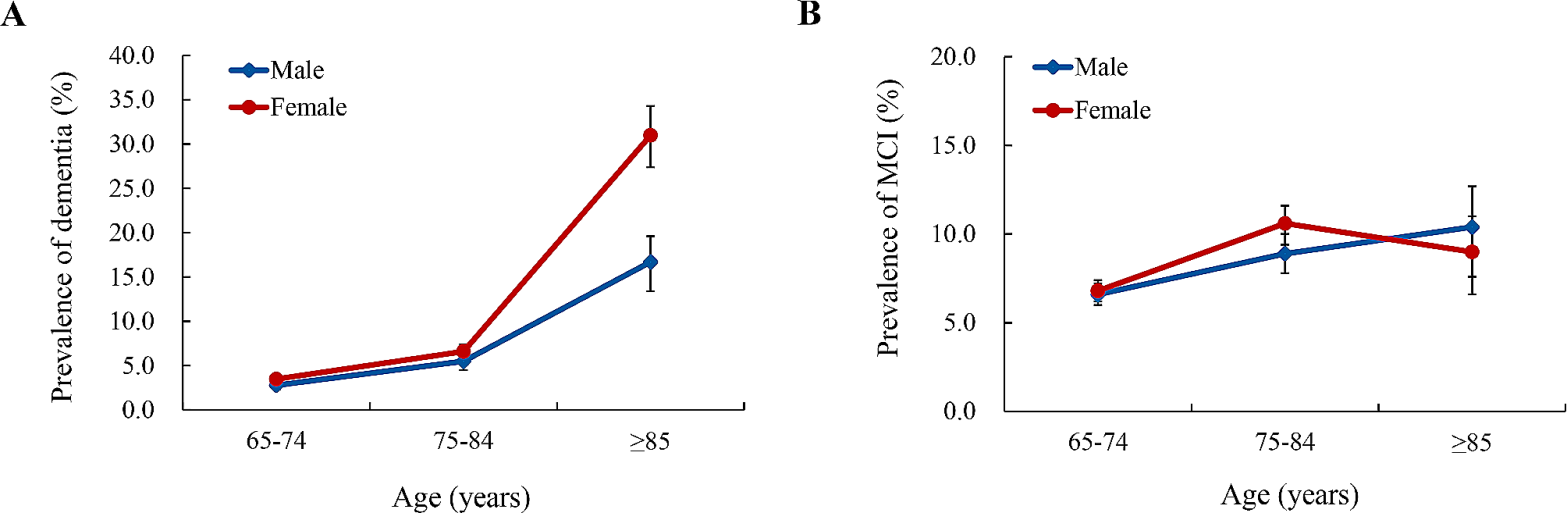
Prevalence of dementia and MCI, stratified by sex and age group. Error bars represent 95% confidence intervals (CIs). MCI, mild cognitive impairment

The overall prevalence of dementia was estimated to be 5.4% (95% CI, 5.1–5.7). The prevalence of dementia increased with age, ranging from 3.2% (95% CI, 2.9–3.5) in the 65–74 years group to 24.5% (95% CI, 22.2–27.0) in the ≥ 85 years group (Table 2; Fig. 1). Furthermore, females, especially those in the ≥ 85 years group, demonstrated a higher prevalence of dementia than males (Table 2; Fig. 2A). The overall prevalence of MCI was 7.7% (95% CI, 7.4–8.1). Across age groups, the prevalence of MCI at 64–74 years, 75–84 years, and ≥ 85 years was 6.7%, 9.8%, and 9.6%, respectively. The results also showed that the prevalence of MCI among females aged 75–84 years was higher than that among males. In contrast, the prevalence among females aged ≥ 85 years was lower than that among males (Fig. 2B).

**Table 2:** Prevalence (%) and 95% CI of dementia and mild cognitive impairment by demographics

	Dementia	MCI
Overall	5.4 (5.1–5.7)	7.7 (7.4–8.1)
Age group		
65–74 years	3.2 (2.9–3.5)	6.7 (6.3–7.1)
75–84 years	6.1 (5.5–6.8)	9.8 (9.0-10.6)
≥ 85 years	24.5 (22.2–27.0)	9.6 (8.1–11.4)
Gender		
Male	4.5 (4.11–4.9)	7.5 (6.9-8.0)
Female	6.2 (5.7–6.6)	8.0 (7.5–8.5)
Educational level		
< 1 years	8.8 (7.6–10.1)	5.6 (4.6–6.7)
1–6 years	5.6 (5.0-6.3)	5.3 (4.7-6.0)
> 6 years	4.8 (4.4–5.2)	9.0 (8.5–9.5)
Hypertension		
No	3.6 (3.3-4.0)	6.3 (5.9–6.8)
Yes	7.6 (7.1–8.2)	9.5 (8.9–10.1)
Diabetes		
No	4.4 (4.1–4.8)	7.2 (6.8–7.6)
Yes	9.3 (8.4–10.3)	9.8 (8.9–10.8)

Table 3 shows the outcomes of the multivariable-adjusted logistic regression analyses. We found that female sex was associated with an increased risk of dementia. Compared with individuals aged 64–74 years, the OR for dementia was 2.09 (95% CI, 1.80–2.42) among those aged 75–84 years; the OR significantly increased to 10.42 (95% CI, 8.80–12.34) among those aged ≥ 85 years. Furthermore, the presence of hypertension (OR, 2.33; 95% CI, 2.05–2.65) or DM (OR, 2.32; 95% CI, 2.02–2.66) was identified as a significant risk factor for dementia. The results indicated that older age, female sex (OR, 1.22; 95% CI, 1.10–1.36), hypertension (OR, 1.66; 95% CI, 1.49–1.84), and DM (OR, 1.51; 95% CI, 1.33–1.70) were associated with an increased risk of MCI. Compared with individuals who had no formal education, the OR for MCI was 1.83 (95% CI, 1.49–2.25) among those who had received > 6 years of education. The 2,635 participants with dementia or MCI identified through screening were advised to visit the general hospital for further diagnosis and treatment. However, during follow-up visits, we observed that only 5.5% (144/2635) of the older adults with dementia or MCI opted to pursue further diagnosis and treatment at the hospital.

**Table 3:** Odd ratios for dementia and MCI

	Dementia (*n* = 1,082)		MCI (*n* = 1,553)	
	Unadjusted OR (95% CI)	Adjusted OR (95% CI)	Unadjusted OR (95% CI)	Adjusted OR (95% CI)
Age, years	1.12 (1.11–1.13)^***^	-	1.04 (1.03–1.04)^***^	-
Age group				
65–74 years	1 (ref)	1 (ref)	1 (ref)	1 (ref)
75–84 years	2.05 (1.77–2.37)^***^	2.09 (1.80–2.42)^***^	1.57 (1.41–1.76)^***^	1.60 (1.43–1.79)^***^
≥ 85 years	10.50 (8.94–12.33)^***^	10.42 (8.80-12.34)^***^	2.28 (1.86–2.79)	2.23 (1.82–2.73)^***^
Gender				
Male	1 (ref)	1 (ref)	1 (ref)	1 (ref)
Female	1.41 (1.24–1.60)^***^	1.44 (1.26–1.65)^***^	1.13 (1.08–1.25) ^***^	1.22 (1.10–1.36) ^***^
Educational level				
< 1 years	1 (ref)	1 (ref)	1 (ref)	1 (ref)
1–6 years	0.62 (0.51–0.75)^***^	0.87 (0.70–1.08)	0.62 (0.51–0.76)^***^	1.00 (0.79–1.26)
> 6 years	0.54 (0.45–0.65)^***^	0.95 (0.78–1.15)	0.57 (0.50–0.66)^***^	1.83 (1.49–2.25)^***^
Hypertension				
No	1 (ref)	1 (ref)	1 (ref)	1 (ref)
Yes	2.27 (2.00-2.58)^***^	2.33 (2.05–2.65)^***^	1.62 (1.46–1.80)^***^	1.66 (1.49–1.84)^***^
Diabetes				
No	1 (ref)	1 (ref)	1 (ref)	1 (ref)
Yes	2.29 (2.01–2.61)^***^	2.32 (2.02–2.66)^***^	1.49 (1.32–1.68)^***^	1.51 (1.33–1.70)^***^

Note: OR, odds ratio; MCI, mild cognitive impairment. ^***^p < 0.001; ^**^p < 0.01; ^*^p < 0.05

## Discussion

Our study was based on a government project titled “Bring Tangible Benefits to the People” of Xiamen City, Fujian Province, China. The project focused on residents aged ≥ 65 years and aimed to explore the current status, including the prevalence and risk factors, of dementia and MCI in Xiamen City. According to the 2021 census, among a total population of 5.3 million in Xiamen city, 367,000 people were aged ≥ 65 years. This study enrolled 20,070 participants, constituting 5.5% of the population aged ≥ 65 years. Our results showed that the overall prevalence of dementia in Xiamen was 5.4%. A previous large-sample report for Chinese individuals aged ≥ 65 years involving 32,552 respondents in 2019 and 10,276 in 2014 indicated a 5.60% and 5.14% prevalence of dementia, respectively [18, 19]. Another study encompassing 46,011 Chinese respondents aged ≥ 60 years in 2020 reported a 6.0% prevalence of dementia [8]. Thus, the prevalence of dementia in our survey was consistent with previous findings among Chinese individuals. However, compared with the prevalence of dementia in other countries, our findings showed a significantly lower prevalence than those in Japan (11.3%) [20], Latin America and the Caribbean (10.66%) [21], South Korea (9.20%) [22], the United States (8.5%) [23], and India (7.4%) [24]. Conversely, it was higher than those reported in Benin (3.2%) [25] and Portugal (3.65%) [26]. The prevalence of dementia varies greatly among countries. Several factors were considered to contribute to these variations, such as genetic factors, sample size, diverse diagnostic criteria, and environmental risks. Moreover, we identified 1,553 individuals with MCI, yielding a prevalence of 7.7%. The prevalence of MCI ranged from 5.0 to 36.7% in different regions among individuals aged ≥ 60 years [27]. Two reports on the Chinese population aged ≥ 60 years indicated that the prevalence of MCI was 14.7% in 2018 and 15.5% in 2020 [8, 11]. Another report showed that the prevalence of MCI was 19.5% (23.4% in rural and 16.8% in urban areas) among Chinese individuals aged ≥ 65 years [28]. Our study enrolled an older and relatively less educated population. However, the estimated prevalence of dementia and MCI was lower than that of other populations outside China. This may attribute to the combined effect of multiple factors. First, it may be related to the dietary habits. The diet of the population in Southeast China is rich in vegetables, fruits, and fish, as well as low in salt and fat. Growing evidence suggests that diets rich in vegetables and low in salt and fat can be protective against cognitive decline [29]. Second, genetic factors may have contributed to these results.

Our results showed that two unmodifiable risk factors, increasing age and female sex, were associated with dementia and MCI, similar to those of another study [30]. Additionally, two modifiable risk factors, hypertension and DM, were related to dementia and MCI. Previous reports have also indicated a remarkable increase in the MCI risk among individuals with hypertension and DM [31]. Another meta-analysis suggested that DM resulted in gray matter atrophy and accelerated brain aging [32]. Moreover, hypertension and DM are associated with amyloid-β burden and contribute to the pathophysiology of MCI [33]. To date, no effective medical treatment is available for MCI. Therefore, interventions targeting modifiable risk factors are particularly crucial for preventing cognitive deterioration. Healthy lifestyles and regular use of medications to manage hypertension and DM were considered to lower the risk of developing cognitive decline [34, 35]. Meanwhile, contrary to previous studies, our results indicated that the OR for MCI in the population that had received > 6 years of education was higher than that in the population that had received no formal education. No direct correlation has been established between education level and cognitive impairment. However, individuals with higher levels of education may possess stronger learning, thinking, and problem-solving abilities. Nonetheless, highly educated individuals may experience more negative affective states, such as anxiety and depression, or have more unhealthy lifestyle habits, which may also increase the risk of cognitive impairment.

While we maintained scientific rigor throughout this study, we acknowledge several limitations. First, despite enrolling over 20,000 older people in Xiamen City, conducting similar research in more cities across Southeast China would enhance the credibility and comprehensiveness of our findings. Second, we did not classify dementia according to its etiology, such as vascular dementia or Alzheimer’s disease. Finally, compared with previous large-scale studies conducted in China, we chose two possible modifiable risk factors for analysis. At the outset of the in-person interviews, we collected the participant’s personal and medical histories, such as hypertension, DM, hyperlipidemia, heart disease, and smoking and drinking habits. However, as the interviews progressed, we discovered through quality analysis that much of the information provided by the participants was unreliable. For instance, the reliability of hyperlipidemia data was compromised due to the lack of recent blood lipid level test results for many participants in hospital records. Additionally, most participants refused to go to the hospital for examination. Therefore, we utilized portable blood pressure and glucose monitors to test the blood pressure and blood glucose levels, respectively, of the participants for the determination of hypertension and diabetes diagnoses. Furthermore, due to the older age of the participants, the information regarding their lifestyle issues was often inaccurate and ambiguous. To ensure the reliability and authenticity of the data, we chose to focus our data collection and analysis on stable and reliable factors, such as hypertension and DM.

During follow-up visits, we discovered that only 5.5% (144/2635) of the older adults with dementia or MCI opted to pursue further diagnosis and treatment at the hospital. This low referral rate among positive participants may be attributed to several reasons. One possible reason is the impact of the coronavirus disease pandemic, as patients and their family members may have feared contracting the virus when visiting the hospital. Some individuals may consider amnesia or cognitive dysfunction to be a normal aging process; hence, they were reluctant to seek medical attention. Additionally, education and knowledge about dementia and MCI have not been widely disseminated, posing challenges for family members in recognizing the detrimental effects and prognosis of cognitive impairment. Recommendations have been made to improve the referral rate of patients with cognitive impairment from the community. First, specialists in cognitive disorders from general hospitals should conduct more lectures and presentations on dementia or MCI to train doctors in the community. Moreover, government departments should implement more science education initiatives in the community to raise public awareness of cognitive disorders.

## Conclusions

The estimated prevalence of dementia and MCI was 5.4% and 7.7%, respectively, among individuals aged ≥ 65 years in Southeast China. Meanwhile, our results indicated that dementia and MCI share similar risk factors, including older age, female sex, hypertension, and DM. Additionally, only 5.5% of the positive participants opted for referral to the hospital for further diagnosis and treatment during follow-up visits. These findings are crucial for preventing and managing dementia and MCI in China.

## Data Availability

The data are available from the corresponding author upon reasonable request.

## Abbreviations

MCI: Mild cognitive impairment
MMSE: Mini-Mental State Examination
CDR: Clinical Dementia Rating
ADL: Activities of Daily Living
DM: Diabetes Mellitus
OR: Odds ratio
CI: Confidence interval
